# CircRNA Cdr1as functions as a competitive endogenous RNA to promote hepatocellular carcinoma progression

**DOI:** 10.18632/aging.102312

**Published:** 2019-10-01

**Authors:** Yang Su, Xiurui Lv, Wei Yin, Lingling Zhou, Yilin Hu, Ang Zhou, FuZhen Qi

**Affiliations:** 1 Department of Hepatobiliary and Pancreatic Surgery, The Affiliated Huaian No.1 People’s Hospital of Nanjing Medical University, Huaian, Jiangsu, China; 2 State Key Laboratory of Reproductive Medicine, Center for Global Health, Key Laboratory of Modern Toxicology of Ministry of Education, School of Public Health, Nanjing Medical University, Nanjing, Jiangsu, China; 3 Department of Pediatric Surgery, Children’s Hospital of Nanjing Medical University, Nanjing, Jiangsu, China; 4 Department of General Surgery, The Affiliated Huai'an Hospital of Xuzhou Medical University, Huaian, Jiangsu, China; 5 Research Center of Clinical Medicine, Nantong University Affiliated Hospital, Nantong, Jiangsu, China

**Keywords:** hepatocellular carcinoma, circRNA Cdr1as, competing endogenous RNAs, exosomes, AFP

## Abstract

Hepatocellular carcinoma (HCC) is one of the most common malignancies worldwide. Recent years, circular RNA (circRNA) have been shown to exert vital functions in the pathological progressions of many diseases. A growing number of evidences have identified the representative function of exosomal circRNAs in the physiological state of donor cells, which further induces cellular responses after captured by recipient cells. However, the contributions of circRNAs to HCC remain largely unknown. *In vitro* and *in vivo* regulatory roles of circRNA Cdr1as in proliferative and migratory abilities of HCC were evaluated by CCK8, EdU, Transwell and tumourigenicity assays, respectively. Results showed circRNA Cdr1as was highly expressed in HCC cell lines and tissues. Overexpression of circRNA Cdr1as greatly accelerated HCC cells to proliferate and migrate. Mechanistically, we found that Cdr1as could promote the expression of AFP, a well-known biomarker for HCC, by sponging miR-1270. Further studies showed exosomes extracted from HCC cells overexpressing circRNA Cdr1as accelerated the proliferative and migratory abilities of surrounding normal cells. In all, circRNA Cdr1as serves as a ceRNA to promote the progression of HCC. Meanwhile, it is directly transferred from HCC cells to surrounding normal cells *via* exosomes to further mediate the biological functions of surrounding cells.

## INTRODUCTION

Hepatocellular carcinoma (HCC) is one of the most common malignancies all over the world [1–3]. Although it has long been highly prevalent in Asia and Africa, it was relatively less common in the Western world. However, over the past decades HCC incidence has doubled in the United Kingdom and tripled in the United States [4, 5]. Largely because of the propensity for metastasis, the five-year survival rate of patients with HCC remains poor [6–9]. Identifying prognostic biomarkers and treatment targets for HCC is of paramount importance. Some genes have already been identified to participate in the pathogenesis of HCC, including CYP1A1, p53, PTEN, ALDH2, EPHX1, etc [10–14]. Besides, some non-coding RNAs (ncRNAs) have also been proved to regulate the biological processes of HCC, including miRNA-302b, LncRNA DCST1-AS1 and circADAMTS13 [15–17]. We aimed to explored the pathogenesis of HCC at the epigenetic level.

According to the structure of ncRNAs, they can be divided into linear ncRNAs and circRNAs [18–20]. Compared with linear ncRNAs, circRNAs were less reported and were considered as by-products of mis-splicing [21]. Nevertheless, recent studies have shown the crucial roles of circRNAs in the pathological processes of various diseases, such as immune system diseases, digestive diseases and urinary system diseases [22–25]. For example, Chen L et al. revealed that circRNA_100290 is able to mediate the progression of oral cancer by sponging the miR-29 family [26]. CircRNA Cdr1as (ID: hsa_circ_0001946 in circBase) is located at chrX:139865339-139866824. CDR1 is the associated-gene symbol of circRNA Cdr1as. It is reported that circRNA Cdr1as acts as a risk factor in HCC [27]. The specific regulatory mechanism of circRNA Cdr1as in HCC still requires for further explorations.

Exosomes are the origin of the inner circulation of extracellular vesicles and are incorporated into components of donor cells, including signaling proteins, RNAs (circRNA or linear RNA), transcriptional regulators, DNAs and lipids [28, 29]. These substrates can be taken to adjacent or distant cells, thereafter mediating recipient cell functions [30–32]. Exosomal circRNAs are reported to be important in tumors [33, 34]. In this study, we examined exosomal circRNA Cdr1as level in 293T and HCC cells, which was remarkably upregulated in HCC cells. So far, evidences on the biological functions of exosomal circRNA Cdr1as in HCC are lacked, which are fully explored in this paper. We aim to elucidate whether exosomal circRNA Cdr1as could be a cellular link to promote the progression of HCC.

To sum up, we verified that circRNA Cdr1as sponged miR-1270 to mediate AFP level in HCC cells, serving as a competing endogenous RNAs (ceRNA). Moreover, it is directly transferred from HCC cells to surrounding normal cells *via* exosomes to further mediate the biological functions of surrounding normal cells.

## RESULTS

### CircRNA Cdr1as in HCC cell lines

**Figure 1:**
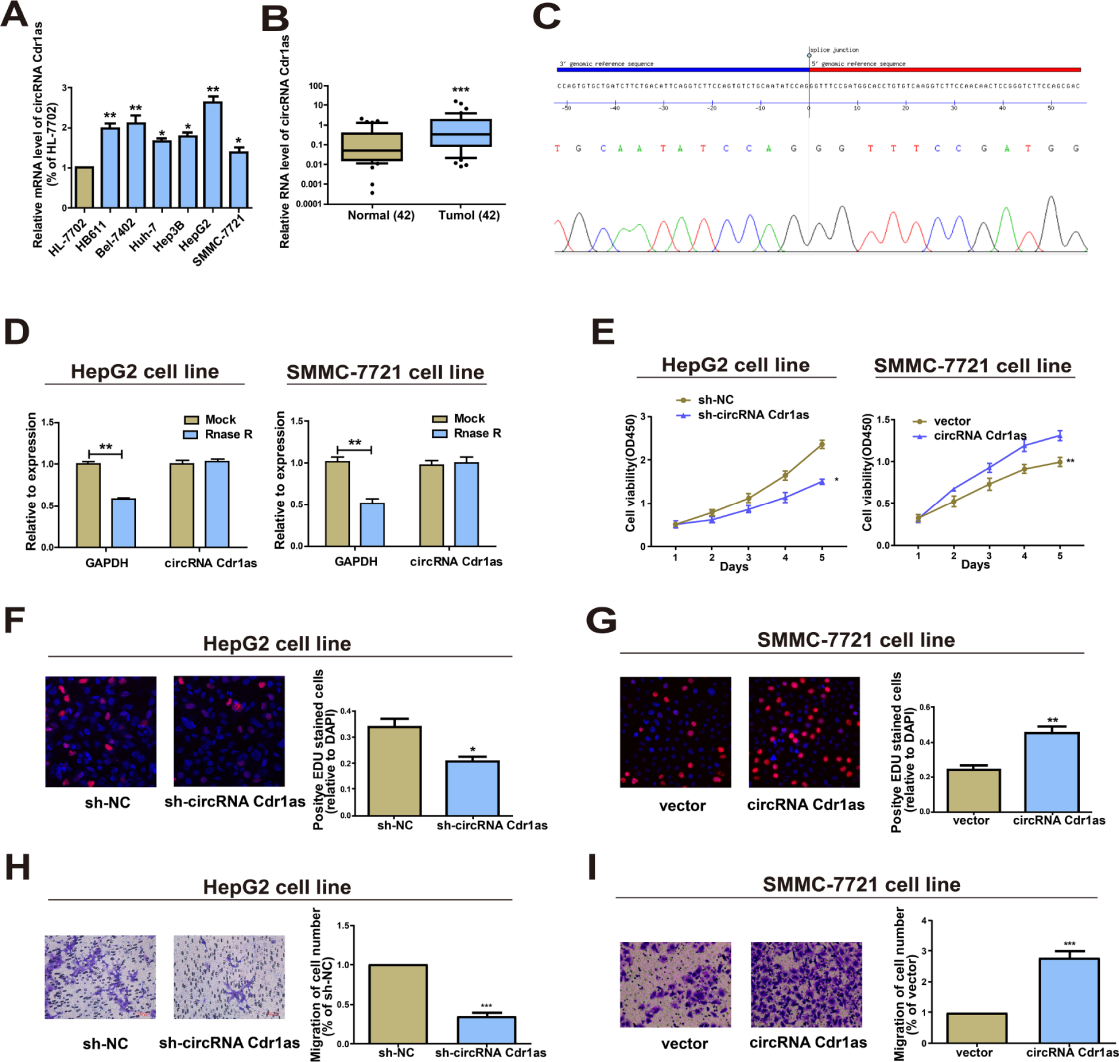
**Functions of circRNA Cdr1as in HCC cell lines.** (**A**) Expression level of circRNA Cdr1as remained higher in HCC cell lines (SMMC-7721, Bel-7402, HepG2, Hep3B, Huh-7, HB611) than human normal liver cell line HL-7702 detected by qRT-PCR. (**B**) qRT-PCR detection of the relative expression of circRNA Cdr1as in paired HCC tumor and paired para-carcinoma tissues (n=42). (**C**) The sequence of circRNA Cdr1as in circBase (upper panel) was consistent with the result of Sanger sequencing (lower panel). (**D**) CircRNA Cdr1as was resistant to RNaseR digestion in HCC cell lines. (**E**) CCK8 assay showed proliferation of HepG2 and SMMC-7721 cells transfected with circRNA Cdr1as shRNA or overexpression vector. (**F**, **G**) EdU assay showed proliferation of HepG2 and SMMC-7721 cells transfected with circRNA Cdr1as shRNA or overexpression vector. (**H**, **I**) Transwell migration assay showed that down-regulation of circRNA Cdr1as inhibited the migration of HepG2 cells, and overexpression of circRNA Cdr1as promoted the migration of SMMC-7721 cells. Photographs were taken under an optical microscope with a magnification of 200×. Results were presented as mean ± SD. *P<0.05, **P<0.01, ***P<0.001. All of the experiments were performed in triplicate.

CircRNA Cdr1as was upregulated in HCC tissues through analyzing the GSE97332 dataset. Identically, circRNA Cdr1as was also highly expressed in HCC cells relative to HL-7702 cell line (Figure 1A). In particular, HepG2 cells had the highest expression, and SMMC-7721 cells had the lowest expression of circRNA Cdr1as, and were chosen for subsequent experiments. Furthermore, the results of circRNA Cdr1as levels in HCC tumors and paired adjacent nonmalignant tissues showed significantly higher circRNA Cdr1as in HCC tumor tissues (Figure 1B). Expression of circRNA Cdr1as in 42 HCC cancer tissues was detected by qRT-PCR. HCC tumor tissues were divided into high circRNA Cdr1as group (n=21) and low circRNA Cdr1as group (n=21) according to the median value. Then we found that low expression of circRNA Cdr1as in the tumor tissues was associated with tumor diameter, serum α-fetoprotein (AFP) and tumor satellite, but not with other clinicopathological features including gender, age, liver function (Child-Pugh stage), grade of differentiation, hepatocirrhosis or HBV infection (Supplementary Table 1). Through sanger sequencing, we confirmed that the circRNA Cdr1as sequence amplified by the primer was identical to its sequence in circbase (Figure 1C). RNase R digestion was conducted to verify the circular characteristics of circRNA Cdr1as. The data showed that circRNA Cdr1as was resistant to RNase R digestion due to the lack of a free 3’terminus (Figure 1D).

### Overexpression of circRNA Cdr1as accelerated HCC cells to proliferate and migrate

Transfection of circRNA Cdr1as shRNA in HepG2 cells or overexpression vector in SMMC-7721 cells sufficiently downregulated or upregulated circRNA Cdr1as level (Supplementary Figure 1A). Knockdown of circRNA Cdr1as markedly inhibited proliferative and migratory abilities in HepG2 cells as Cell Counting Kit-8 (CCK8), 5-Ethynyl-2’-deoxyuridine (EdU) assay and Transwell assay indicated (Figure 1E, 1F and 1H). Conversely, circRNA Cdr1as overexpression in SMMC-7721 cells yielded the opposite trends (Figure 1E, 1G and 1I). In short, these results revealed that circRNA Cdr1as promoted HCC cells to proliferate and migrate.

### CircRNA Cdr1as directly interacts with miR-1270

**Figure 2:**
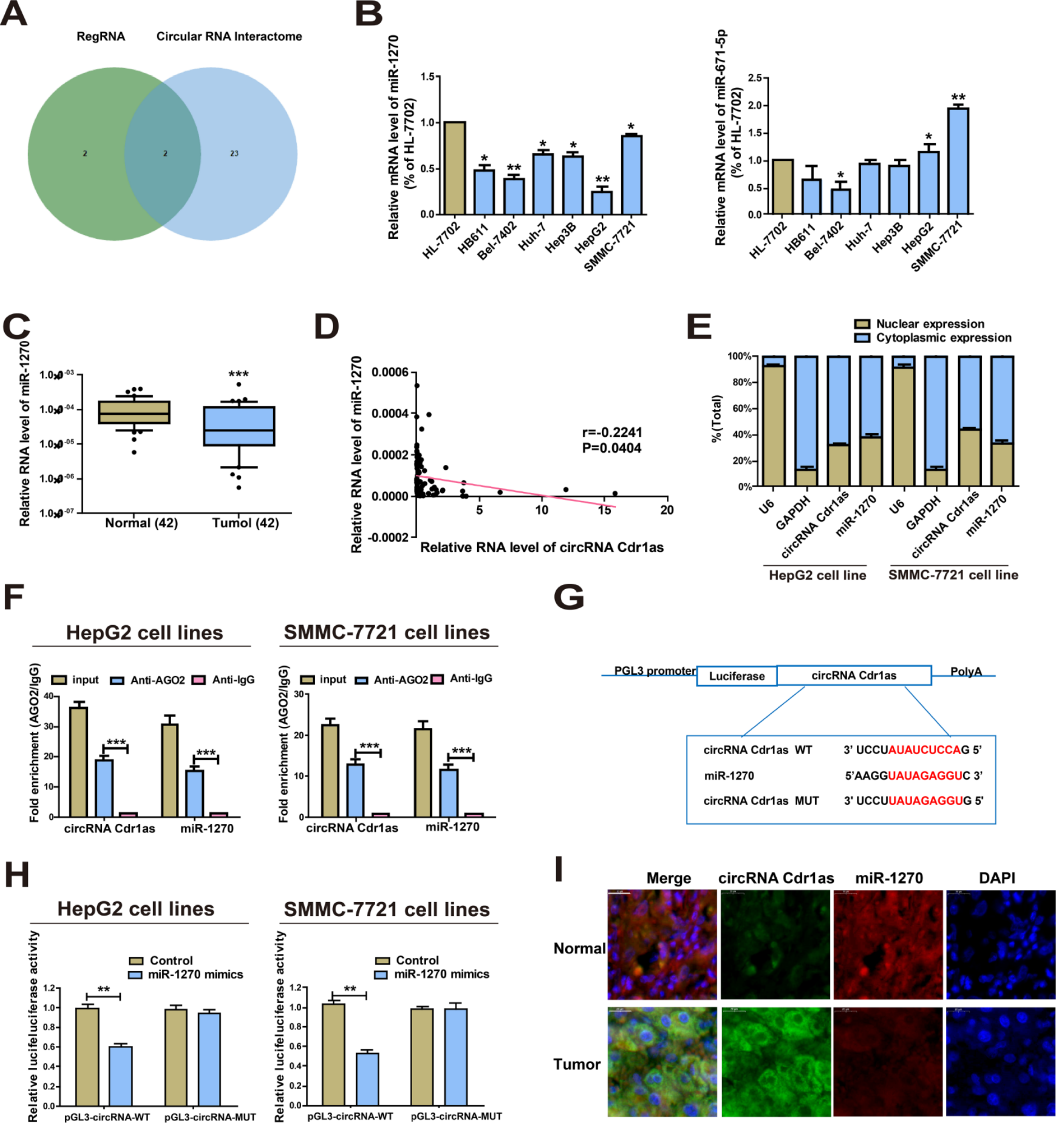
**CircRNA Cdr1as directly interacts with miR-1270.** (**A**) Prediction results on RegRNA 2.0 and CircInteractome. (**B**) Expressions of miR-1270 and miR-671-5p in HCC cells and HL-7702 cells. MiR-1270 level was significantly reduced in HCC cells compared to HL-7702 cells. (**C**) qRT-PCR detection of the relative expression of miR-1270 in paired HCC tumor and paired para-carcinoma tissues (n=42). (**D**) Correlation between circRNA Cdr1as and miR-1270 in HCC samples. (**E**) Expressions of circRNA Cdr1as and miR-1270 in the nucleus and cytoplasmic fractions of HepG2 and SMMC-7721 cells were analyzed by qRT-PCR. (**F**) RIP experiment confirmed the binding relationships of circRNA Cdr1as and miR-1270 in HepG2 and SMMC-7721 cells. CircRNA Cdr1as and miR-1270 levels were detected using qRT-PCR. (**G**) Bioinformatics evidence for the binding of miR-1270 to the 3'-UTR of circRNA Cdr1as. (**H**) The luciferase activity in HepG2 and SMMC-7721 cells after co-transfection of plasmid (pGL3-circRNA Cdr1as-WT or pGL3-circRNA Cdr1as-MUT) and miRNA-1270 mimics tested by dual-luciferase reporter gene assay. (**I**) MiR-1270 co-localized with circRNA Cdr1as in HCC tissues or normal liver tissues was detected by FISH. Results were presented as mean ± SD. *P<0.05, **P<0.01, ***P<0.001. All of the experiments were performed in triplicate.

The specific mechanism of circRNA Cdr1as in mediating HCC cell behaviors was the research focus in the following. Given that circRNA Cdr1as is predominantly located in the cytoplasm, we hypothesized that it may be a ceRNA to exert the biological function. Based on the predicted bioinformatics on RegRNA 2.0 (http://regrna2.mbc.nctu.edu.tw/) and CircInteractome (https://circinteractome.nia.nih.gov/), there were 4 and 25 miRNAs that were highly matched to the circRNA Cdr1as 3'UTR, respectively. By intersection of them, miR-1270 and miR-671-5p were found to be the most matched miRNAs (Figure 2A, Supplementary Table 2). Converse to circRNA Cdr1as, miR-1270 was lowly expressed in HCC cells. However, miR-671-5p did not exert differential expression in HCC cell lines and normal liver cell line (Figure 2B). Then, the results of miR-1270 levels in HCC tumors and paired para-carcinoma normal tissues showed significantly lower miR-1270 in HCC tumor tissues (Figure 2C). Furthermore, we found that miR-1270 expression was inversely correlated with circRNA Cdr1as (Figure 2D). It is well known that the subcellular localization of circRNA determines its function. Here, we isolated cytoplasm and nuclei of HCC cells, and qRT-PCR data revealed that 68.5% and 56.5% of circRNA Cdr1as was distributed in the cytoplasmic fractions of HepG2 and SMMC-7721 cells respectively (Figure 2E). It is believed that circRNA Cdr1as may mediate the pathogenesis of HCC at post-transcriptional level. MiRNA distributes in the cytoplasm, which is a component of the RNA-induced silencing complex (RISC) containing Ago2. Ago2 is required for miRNA-mediated gene silencing. In this study, we analyzed if circRNA Cdr1as and miR-1270 contained the same RISC and performed (RNA Binding Protein Immunoprecipitation) RIP assay in HCC cells. It is shown that circRNA Cdr1as and miR-1270 were enriched in Ago2-containing miRNAs than IgG control (Figure 2F). To further explore the binding between circRNA Cdr1as and miR-1270, we constructed circRNA Cdr1as-WT and circRNA Cdr1as-MUT for performing dual-luciferase reporter gene assay (Figure 2G). HCC cells were co-transfected with circRNA Cdr1as-WT/circRNA Cdr1as-MUT and miR-1270 mimics/negative control, respectively. As the data revealed, luciferase intensity markedly decreased in cells co-transfected with circRNA Cdr1as-WT and miR-1270 mimics, confirming the binding between circRNA Cdr1as and miR-1270 (Figure 2H). MiR-1270 co-localized with circRNA Cdr1as in HCC tissues or normal liver tissues was examined by FISH. Compared with normal liver tissues, circRNA Cdr1as level in HCC tissues was elevated, and meanwhile, miR-1270 level was reduced. Interestingly, no matter of HCC tissues and normal liver tissues, subcellular distribution of circRNA Cdr1as and miR-1270 was consistent (Figure 2I). All the above demonstrated that circRNA Cdr1as directly bound to miR-1270 in HCC.

### circRNA Cdr1as accelerated HCC development by repressing miR-1270

**Figure 3:**
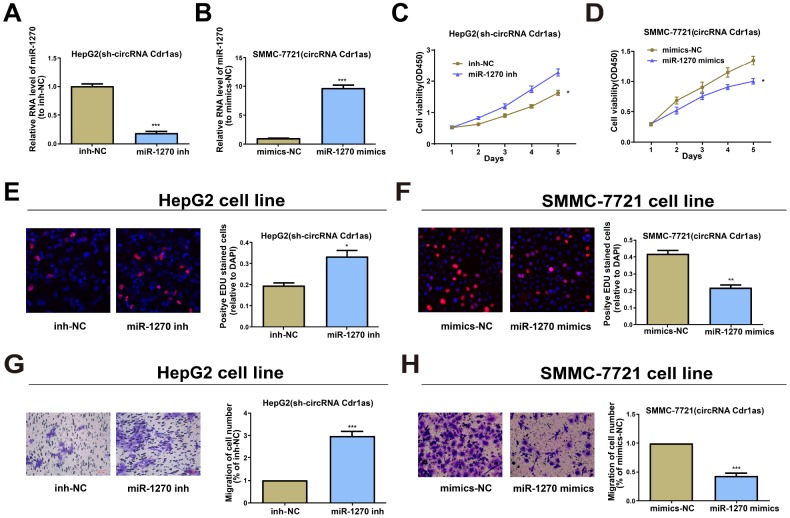
**Effects of circRNA Cdr1as in HCC cells are reversed by miR-1270 to some extent.** (**A**) Relative expression of miR-1270 in circRNA Cdr1as-repressed HepG2 cells treated with miR-1270/NC inhibitor through qRT-PCR. (**B**) Relative expression of miR-1270 in circRNA Cdr1as-overexpressed SMMC-7721 cells treated with miR-1270/NC mimics through qRT-PCR. (**C**, **E**) Cell proliferation capacities of circRNA Cdr1as-repressed HepG2 cells with or without suppression of miR-1270 are analyzed. (**D**, **F**) Cell proliferation capacities of circRNA Cdr1as-overexpressed SMMC-7721 cells with or without upregulation of miR-1270 are analyzed. (**G**) Cell migration capacities of circRNA Cdr1as-repressed HepG2 cells with or without suppression of miR-1270 are analyzed. (**H**) Cell migration capacities of circRNA Cdr1as-overexpressed SMMC-7721 cells with or without upregulation of miR-1270 are analyzed. Data are the mean ± SEM. *P<0.05, **P<0.01, ***P<0.001.

To ascertain that the role of circRNA Cdr1as required miR-1270 reduction in a direct manner, miR-1270 inhibitor was transfected into circRNA Cdr1as-repressed HepG2, and the observably reduced miR-1270 was discovered by qRT-PCR (Figure 3A). Meanwhile, miR-1270 mimics was transfected into circRNA Cdr1as-overexpressed SMMC-7721 cells, and the observably elevated miR-1270 was discovered by qRT-PCR (Figure 3B). A foregone conclusion was obtained from CCK8 and EDU assays that the proliferation capacities of circRNA Cdr1as-repressed HepG2 cells were promoted by miR-1270 suppression (Figure 3C and 3E). While, overexpressing miR-1270 was proved to evidently inhibit the proliferation capacities of circRNA Cdr1as-overexpressed SMMC-7721 cells (Figure 3D and 3F). The similar results were observed in cell migration assays (Figure 3G and 3H). The above discovery implies that miR-1270 participates in the carcinogenic process regulated by circRNA Cdr1as in HCC cells.

### CircRNA Cdr1as regulated AFP, the target gene of miR-1270

**Figure 4:**
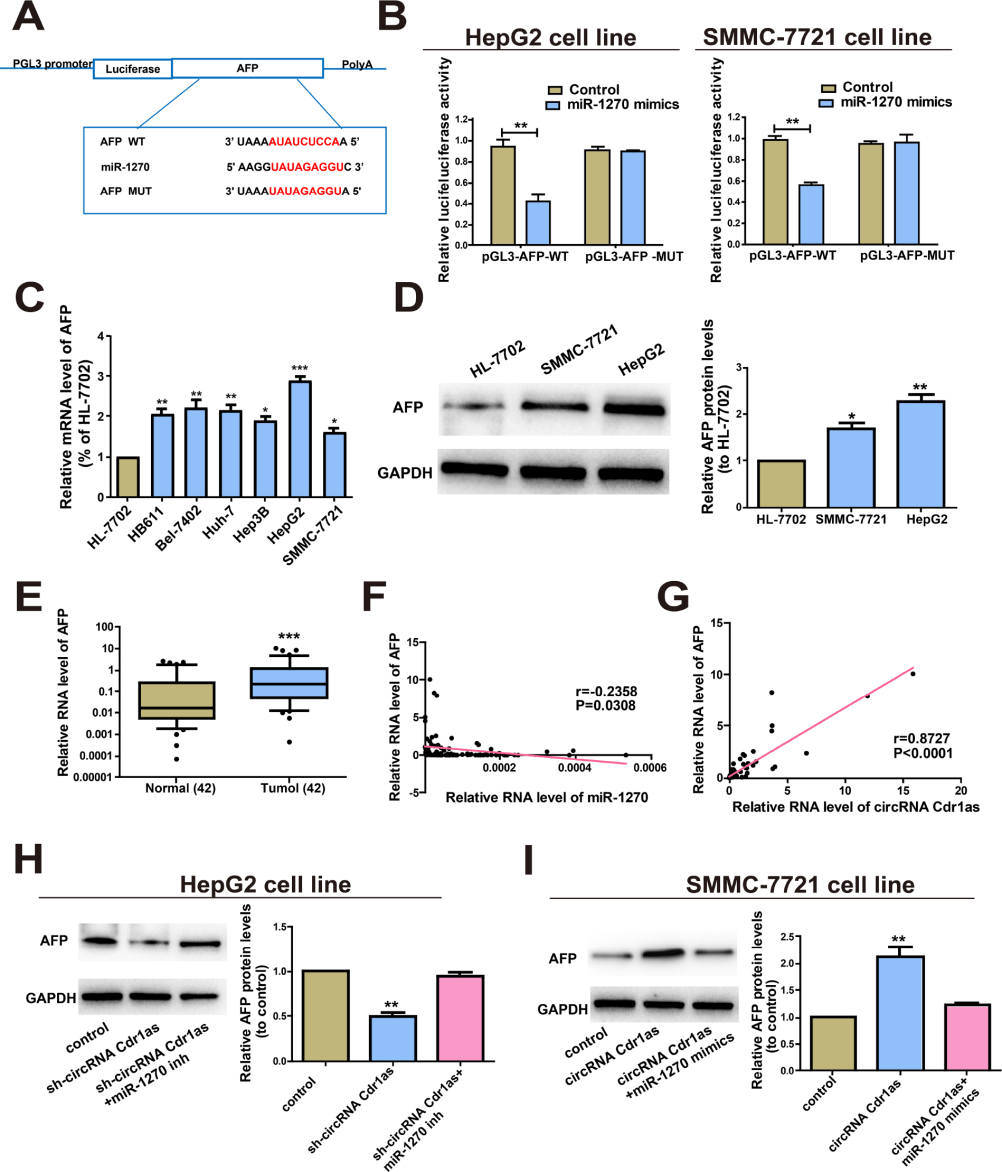
**CircRNA Cdr1as regulated AFP, the target gene of miR-1270.** (**A**) Putative binding site of miRNA in the AFP sequence. The putative miRNAs recognition sites were cloned downstream of the luciferase gene and named pGL3-AFP-WT. (**B**) The luciferase activity in HepG2 and SMMC-7721 cells after co-transfection of plasmid (pGL3-AFP-WT or pGL3-AFP-MUT) and miRNA-1270 mimics was detected by dual-luciferase reporter gene assay. (**C**) Relative expression of AFP in HCC cell lines compared to control human normal liver cell line HL-7702. (**D**) Protein levels of AFP in human normal liver cell line HL-7702, HepG2 and SMMC-7721 cell lines. (**E**) qRT-PCR detection of the relative expression of AFP in paired HCC tumor and paired para-carcinoma tissues (n=42). (**F**) Correlation between miR-1270 and AFP in HCC samples. (**G**) Correlation between circRNA Cdr1as and AFP in HCC samples. (**H**) After HepG2 cells were treated with circRNA Cdr1as shRNA (with inhibitor NC or with miR-1270 inhibitors), Western blotting analysis were adopted to measure the AFP protein level, with GAPDH as a control. (**I**) SMMC-7721 cells were transfected with circRNA Cdr1as overexpression lentiviral vector (with mimics-NC or with miR-1270 mimics), and Western blotting was adopted to detect the AFP protein expression level compared with the control. MiR-1270 inh/mimics means transfection with miR-1270 inhibitor/mimics, sh-circRNA Cdr1as/circRNA Cdr1as means transfection with circRNA Cdr1as shRNA/circRNA Cdr1as overexpressing vector. Results were presented as mean ± SD. *P<0.05, **P<0.01, ***P<0.001. All of the experiments were performed in triplicate.

Bioinformatics prediction (miRDB, RegRNA) was conducted and AFP was selected as the target gene of miR-1270. In the same way, dual-luciferase reporter gene assay was carried out to verify the binding between miR-1270 and AFP. AFP-WT and AFP-MUT plasmids were constructed (Figure 4A). Luciferase intensity markedly decreased in HCC cells co-transfected with AFP-WT and miR-1270 mimics (Figure 4B). QRT-PCR data revealed higher level of AFP in HCC cells relative to control (Figure 4C). Western blot analysis yielded the same trend at the protein level of AFP (Figure 4D). Then, the results of AFP levels in HCC tumors and paired para-carcinoma normal tissues showed significantly higher AFP in HCC tumor tissues (Figure 4E). To sum up, the above-mentioned results elucidate that AFP is a target gene of miR-1270.

Furthermore, Bivariate correlation analysis was processed to assess the interactions between circRNA Cdr1as, miR-1270 and AFP in the tissues. According to the analysis, we found that miR-1270 expression was inversely correlated with AFP (Figure 4F) expression in control and HCC tissues. Interestingly, circRNA Cdr1as positively correlated with the levels of AFP (Figure 4G). To elucidate whether circRNA Cdr1as could mediate AFP by sponging miR-1270, we determined AFP level by Western blot. At the beginning, transfection efficacy of miR-1270 mimics and inhibitor were verified (Supplementary Figure 1B). After HepG2 cells were transfected with sh-circRNA Cdr1as, the results revealed that the protein expression level of AFP was evidently reduced, which could be reversed by the addition of miR-1270 inhibitors (Figure 4H). Then SMMC-7721 cells were transfected with circRNA Cdr1as overexpression vector, and later we found that the protein level of AFP expression was notably increased. However, the addition of miR-1270 mimics could reverse this effect (Figure 4I). Briefly, these data imply that circRNA Cdr1as up-regulated the expression of AFP by regulating miR-1270.

### miR-1270/AFP regulatory loop regulated HCC cell behaviors

**Figure 5:**
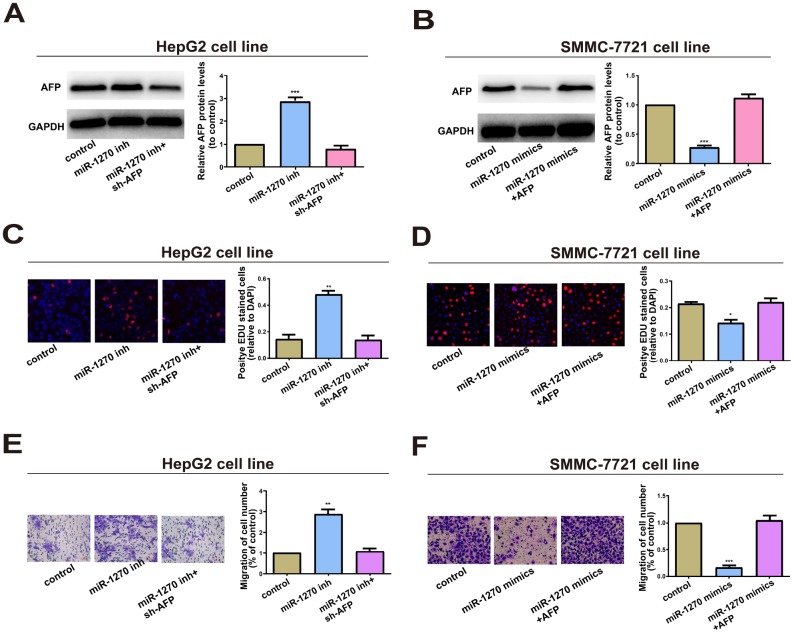
**miR-1270/AFP regulatory loop plays an important role in cell biological Behavior.** (**A**) Western blot analysis of AFP level in HepG2 cells transfected with miR-1270 inhibitor (with or without AFP shRNA). GAPDH was used as a control. (**B**) Western blot analysis of AFP level in SMMC-7721 cells transfected with miR-1270 mimics (with or without AFP overexpression vector). (**C**) EdU assay showed proliferation of HepG2 cells. (**D**) EdU assay showed proliferation of SMMC-7721 cells. (**E**) Transwell assay showed migration of HepG2 cells. (**F**) Transwell assay showed migration of SMMC-7721 cells. Results were presented as mean ± SD. *P<0.05, **P<0.01, ***P<0.001. versus control group. All of the experiments were performed in triplicate.

Whether miR-1270/AFP regulatory loop affects the cell biological behaviors of HCC cells was further explored. Firstly, AFP shRNA and overexpression vector were constructed. Then their transfection efficacy was verified (Supplementary Figure 1C). Transfection of miR-1270 inhibitor markedly upregulated AFP level in HepG2 cells, which was reversed by co-transfection of AFP shRNA (Figure 5A). Conversely, transfection of miR-1270 mimics downregulated AFP level, but was further reversed by co-transfection of AFP overexpression vector (Figures 5B). A series of rescue experiments were designed to explore the role of circRNA miR-1270/AFP regulatory loop in HCC. Compared with the negative control, miR-1270 knockdown in HepG2 cells markedly accelerated proliferative and migratory abilities, which were partially reversed by knockdown of AFP (Figure 5C and 5E). In addition, overexpression of miR-1270 in SMMC-7721 cells inhibited proliferative and migratory abilities relative to control. Upregulation of AFP could partially reverse the regulatory effects of miR-1270 on SMMC-7721 cells (Figure 5D and 5F). In summary, miR-1270/AFP regulatory loop exerted a vital function in HCC cell biological behaviors.

### Knockdown of circRNA Cdr1as inhibited HCC growth *in vivo*

**Figure 6:**
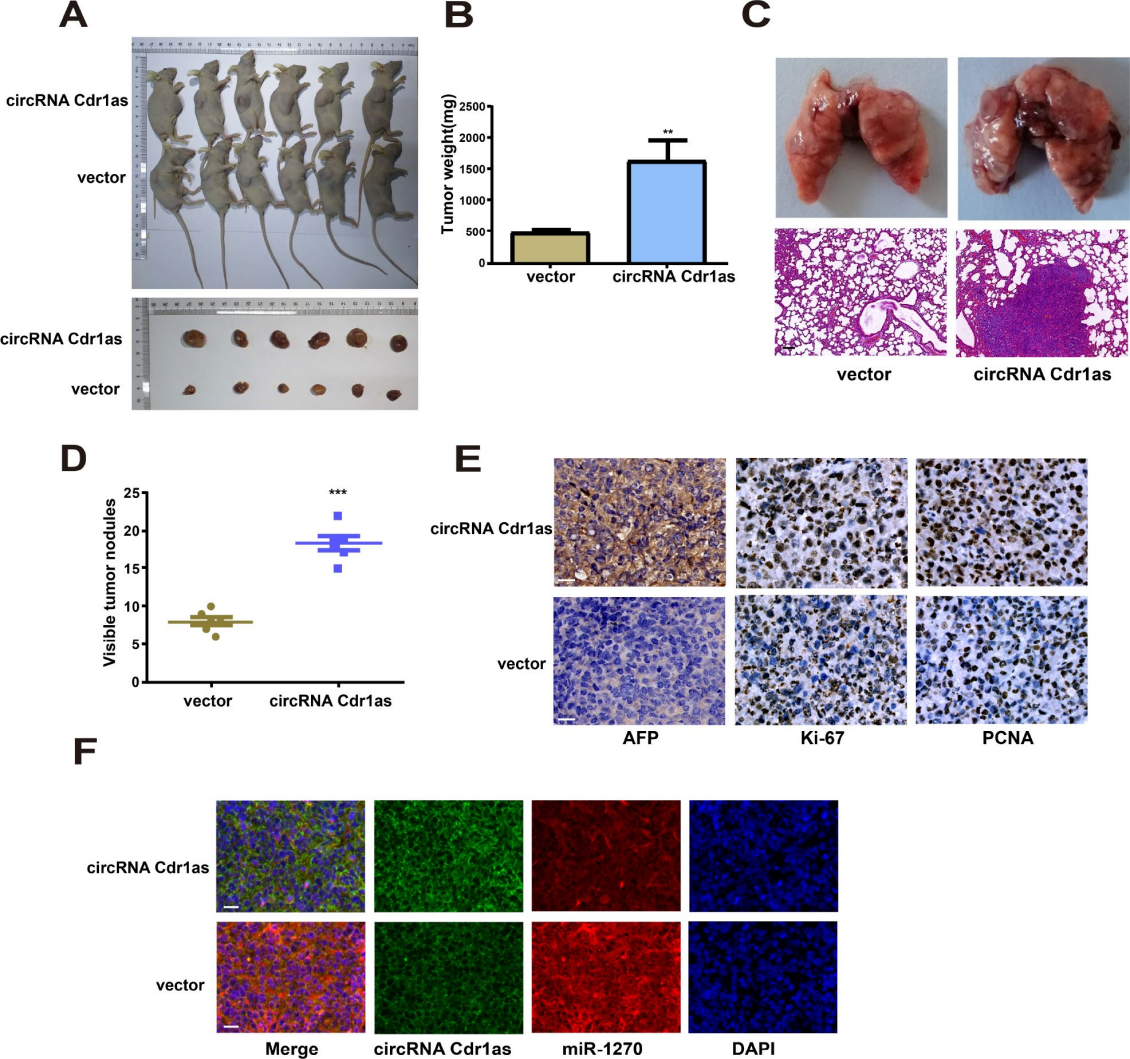
**Upregulation of circRNA Cdr1as in tumors promoted HCC growth *in vivo*.** (**A**) Representative images of xenografts tumor (6 mice per group) in nude mice. (**B**) Xenograft tumor weight. (**C**) Upregulation of circRNA Cdr1as promotes tumor metastasis in vivo (scale bar: 100μm). (Top) Representative bright field images of lungs. (Bottom) Hematoxylin and eosin (HȦE) staining of lung serial sections. (**D**) The number of metastatic nodules. (**E**) Immunohistochemical staining of AFP, Ki-67 and PCNA expression in xenograft tumors (scale bar: 20μm). (**F**) MiR-1270 co-localized with circRNA Cdr1as in xenografts tumor from NC or circRNA Cdr1as overexpressing group was detected by FISH (scale bar: 20μm). Results were presented as mean ± SD. *P<0.05, **P<0.01, ***P<0.001. All of the experiments were performed in triplicate.

To investigate the role of circRNA Cdr1as in the *in vivo* growth of HCC, nude mice were subcutaneously injected with SMMC-7721 cells transfected with circRNA Cdr1as overexpression vector or NC vector. Four weeks later, tumor volume (Figure 6A) and tumor weight (Figure 6B) markedly increased in mice injected with SMMC-7721 cells with circRNA Cdr1as overexpression. To assess the effects of circRNA Cdr1as on the metastasis of HCC in vivo, circRNA Cdr1as overexpression SMMC-7721 cells or NC SMMC-7721 cells were injected via the tail vein into nude mice. Four weeks later, we found that the number and size of metastatic colonies were largely increased on the lung surface in the circRNA Cdr1as overexpression group. (Figure 6C and 6D). Positive expressions of AFP, Ki-67 and PCNA were detected by immunohistochemistry in tumor tissues, which were markedly upregulated by circRNA Cdr1as overexpression (Figure 6E).

MiR-1270 co-localized with circRNA Cdr1as in xenografts tumor was detected by FISH. The data showed increased circRNA Cdr1as level and decreased miR-1270 level in circRNA Cdr1as overexpression group. Identical to population results, subcellular distribution of circRNA Cdr1as and miR-1270 was consistent in nude mouse tumor-bearing tissues (Figure 6F).

### Exosomal circRNA Cdr1as served as a mediator in intercellular communication

**Figure 7:**
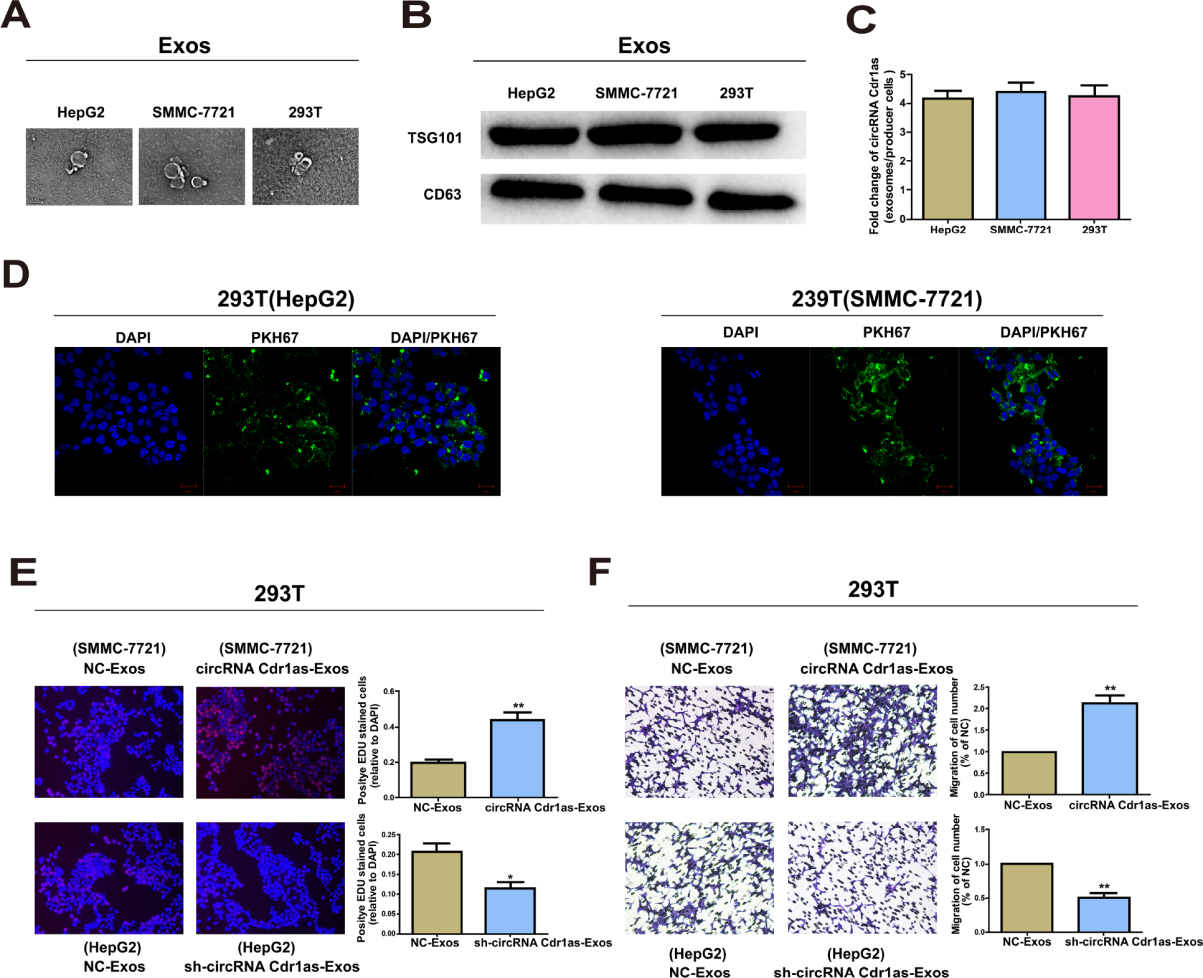
**Exosomal circRNA Cdr1as serve as a mediator in intercellular communication.** Exosomes (Exos) isolated from the medium of 293T, HepG2 and SMMC-7721 cells. (**A**) Micrographs of exosomes isolated from HepG2 (left), SMMC-7721 (middle) and 293T cells (right). (**B**) Western blot analysis of TSG101 and CD63 in exosomes of cell lines. (**C**) qRT-PCR detection of the fold change of circRNA Cdr1as between exos of HepG2, SMMC-7721 and 293T and their producer cells. (**D**) Exos of HepG2 and SMMC-7721 cells were labeled with PKH67; green represents PKH67, and blue represents nuclear DNA staining by DAPI. 293T cells were incubated with exos derived from HepG2 and SMMC-7721 cells for 3 h. (**E**) EdU assays of cell proliferation ability. (**F**) Representative images of migration assays of 293T cells. The number of cells were counted. Results were presented as mean ± SD. *P<0.05, **P<0.01, ***P<0.001. All of the experiments were performed in triplicate.

We explored the existing pattern of extracellular circRNA Cdr1as. Morphology of exosomes was confirmed by TEM (Figure 7A), and surface hallmarks (TSG101 and CD63) were identified by Western blot (Figure 7B). We then found that circRNA Cdr1as expression was remarkably higher in HepG2 and SMMC-7721 cells than in 293T cells (Supplementary Figure 2A). As expected, exosomal circRNA Cdr1as level remained higher in HepG2 and SMMC-7721 cells compared with 293T cells (Supplementary Figure 2B). The data revealed that circRNA Cdr1as level was enriched in exosomes with at least 4-fold higher to that of producer cells (Figure 7C). A green fluorescent marker, PKH67 was used to label exosomes derived from HepG2 and SMMC-7721 cells. After recipient cells (293T cells) were incubated with labeled exosomes derived from HepG2 and SMMC-7721 cells for 3 h, we observed the distribution of PKH67 in the cytoplasm of recipient cells (Figure 7D).

### Effect of exosomal circRNA Cdr1as on surrounding cell phenotype

Exosomes are well-known cellular communication link, which mediate recipient cell function using bioactive factors, including circRNAs [35]. The above results have already illustrated the transfer of circRNA Cdr1as from SMMC-7721 and HepG2 cells into 293T cells *via* exosomes. We then speculated whether exosomal circRNA Cdr1as derived from SMMC-7721 and HepG2 cells could change the biological function of 293T cells. Exosomes contain diverse cargoes, such as transcriptional regulators, various RNA species and DNA [36]. Exosomes extracted from HepG2 cells transfected with circRNA Cdr1as shRNA vector or NC, were named sh-circRNA Cdr1as-Exos or NC-Exos. Meanwhile, exosomes extracted from SMMC-7721 cells transfected with circRNA Cdr1as overexpression vector or NC vector were named circRNA Cdr1as-Exos or NC-Exos. 100 μg/ml of these exosomes were incubated in 293T cells for 24 h. Functional experiments illustrated that compared with negative control, sh-circRNA Cdr1as-Exos could markedly inhibit proliferative and migratory abilities of 293T cells (Figure 7E, 7F). As expected, circRNA Cdr1as-Exos achieved the opposite functions (Figure 7E, 7F). CircRNA Cdr1as level in 293T cells exposed to circRNA Cdr1as-Exos was elevated, which was reduced by exposure to sh-circRNA Cdr1as-Exos (Supplementary Figure 3A and 3B).

**Figure 8:**
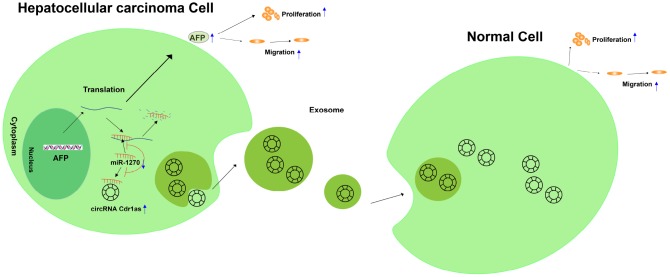
**Graphical abstract of how circRNA Cdr1as promotes hepatocellular carcinoma progression.** A schematic model of circRNA Cdr1as/miR-1270/AFP signaling pathway in hepatocellular carcinoma. circRNA Cdr1as competitively binds to miR-1270, resulting in upregulation of AFP. Furthermore, upregulation of AFP stimulates the migration and proliferation of HCC cell lines. Meanwhile, exosome-transmitted circRNA Cdr1as can stimulate malignant behaviors of surrounding normal cells and finally contributes to the progression of hepatocellular carcinoma.

To sum up, circRNA Cdr1as competitively bound to miR-1270 to upregulate AFP level, thereafter accelerating proliferative and migratory abilities of HCC cells. Meanwhile, exosome-transmitted circRNA Cdr1as stimulated malignant behaviors of surrounding normal cells and finally contributed to the progression of HCC (Figure 8).

## DISCUSSION

The ceRNA hypothesis proposes a new mechanism of RNA interaction [37, 38]. Multiple patterns are existed in transcriptional regulation of genes. MiRNAs are important mediators in biological processes, which are short RNAs with 22 nt in length [39]. They negatively regulate gene expressions by inhibiting translation or directly degrading the target mRNAs [40, 41]. In addition to the regulatory pattern of miRNA-mRNA, some ncRNAs containing miRNA binding sequences could serve as miRNA sponge [42–44]. These ncRNAs further abolish the inhibitory effect of target miRNA on the downstream genes, which is known as the ceRNA hypothesis [45]. Recent studies have shown that various circRNAs are capable of mediating tumor cells to proliferate and migrate as ceRNAs [46–48]. In this paper, we aim to explore whether circRNA Cdr1as could be a ceRNA to influence proliferative and migratory abilities of HCC cells.

CircRNA Cdr1as was highly expressed in HCC cell lines relative to control. Subsequently, we proved the *in vivo* and *in vitro* regulatory effects of circRNA Cdr1as

on proliferative and migratory abilities of HCC. We believed that circRNA Cdr1as was a positive regulator of HCC growth, manifesting as an oncogene. It is crucial to clarify the role of circRNA Cdr1as in the occurrence, progression and metastasis of HCC.

By extracting cytoplasmic and nuclear fractions of HCC cells, circRNA Cdr1as was identified to be mainly distributed in the cytoplasm, suggesting the possibility of circRNA Cdr1as to be a ceRNA. Through RIP, dual-luciferase reporter gene assay and FISH, it is confirmed that circRNA Cdr1as could sponge miR-1270 to upregulate AFP level. So far, relevant researches on the role of miR-1270 in HCC are rarely reported.

The human alpha-fetoprotein (AFP) gene is located in the 4q13.3, and encodes AFP protein. AFP protein is a glycoprotein containing 591 amino acids with a half-life of 5-7 days. Under normal circumstances, AFP is produced by the fetal yolk sac, liver and intestine. Elevated level of AFP is generally used as a biomarker for HCC [49, 50]. AFP is overexpressed in HCC cells [51, 52], and is strongly associated with aggressiveness of cancer cells [53]. Yang T et al. demonstrated that the mRNA level of AFP is upregulated in blood samples of HCC patients, which is a potential hallmark for predicting the tumor metastasis [54]. Yang X et al. suggested that AFP knockdown suppresses growth and induces apoptosis of HepG2 cells [55]. In this study, miR-1270 remained a low level in HCC cells, whereas AFP showed a great abundance. Transfection of circRNA Cdr1as shRNA markedly downregulated AFP level in HCC cells, which was reversed by co-transfection of miR-1270 inhibitor. Meanwhile, miR-1270 overexpression inhibited HCC cells to proliferate and migrate, but were further reversed by overexpression of circRNA Cdr1as or AFP. All the above data demonstrated the effect of circRNA Cdr1as/miR-1270/AFP regulatory loop on the progression of HCC.

Interestingly, circRNA Cdr1as level remained higher in HCC cells and their exosomes, showing four-fold higher than that in producer cells. Hence, circRNA Cdr1as was mainly located in exosomes. Previous studies illustrated the potential of exosomal circRNAs as representative of donor cell condition, which further induce cellular responses after captured by recipient cells [56, 57]. Through morphology examination by TEM, surface marker identification [58, 59] and fluorescence microscopy, we proved that exosomes derived from HCC cells that were labeled with PKH67 could transfer into normal cells. More importantly, circRNA Cdr1as-Exos from HCC cells enhanced circRNA Cdr1as expression and accelerated proliferative and migratory abilities of surrounding normal cells. Hence, our findings suggested that circRNA Cdr1as was directly transferred from HCC cells to surrounding normal cells *via* exosomes and thereafter regulated the biological functions of surrounding normal cells.

In conclusion, circRNA Cdr1as serves as a ceRNA to promote the progression of HCC by sponging miR-1270 to upregulate AFP level. Meanwhile, it is directly transferred from HCC cells to surrounding normal cells *via* exosomes to further stimulate malignant behaviors of surrounding normal cells. Collectively, we demonstrated for the first time that there was a higher expression of circRNA Cdr1as in HCC. Furthermore, our findings also show that exosomal circRNA Cdr1as may be a promoting factor for HCC progression, providing new insights into understanding the pathogenesis of HCC. Finally, this study provided the first evidence for circRNA Cdr1as and exosomal circRNA Cdr1as becoming the potential therapeutic target of HCC, although intensive researches are in sore need to strengthen its clinical value in the future.

## MATERIALS AND METHODS

### Cell culture and transfection

HCC cell lines (SMMC-7721, Bel-7402, HepG2, Hep3B, Huh-7, HB611) and human-derived liver cell line (HL-7702) were purchased from the Type Culture Collection of the Chinese Academy of Sciences (Shanghai, China). Cells were cultured in DMEM (Dulbecco’s Modified Eagle medium, Hyclone, UT, USA) or RPMI 1640 (Roswell Park Memorial Institute, GIBCO, Shanghai, China) containing 10% FBS (fetal bovine serum, Beyotime, Nantong, China), 100 μg/ml streptomycin and 100 IU/ml penicillin (Invitrogen, Carlsbad, CA, USA). They were maintained at 37°C, 5% CO_2_.

Until 50-80% of confluence, cells were infected with 1×10^6^ recombinant lentivirus-transducing units and 6 μg/mL Polybrene (Sigma, Shanghai, China), followed by 2 μg/mL puromycin treatment for 2 weeks. Stably transfected cells were screened by flow cytometry. Transfection vectors were provided by GenePharma Co., Ltd. (Shanghai, China). Transfection of miRNA mimics or inhibitors were conducted using Lipofectamine 3000 (Invitrogen, CA, USA).

### RNA extraction and qRT-PCR

We extracted total RNA from cells with TRIzol reagent (Life Technologies, CA, USA) and were subjected to NanoDrop 2000 Spectrophotometer (Thermo Scientific, Wilmington, DE, USA) for quantification. Qualified RNA was reversely transcribed using the Reverse Transcription Kit (Takara, Tokyo, Japan) and amplified using SYBR Premix Ex Taq (Takara, Tokyo, Japan) on Light Cycler 480 (Roche, BSL, Switzerland). PCR primers were listed in Table 1.

**Table 1:** Sequences of primers for qRT-PCR and miRNA related sequence.

**Name**		**Sequence**
circRNA Cdr1as	Forward	5′-TCAACTGGCTCAATATCCATGTC-3′
	Reverse	5′-ACCTTGACACAGGTGCCAT-3′
miR-1270	Forward	5′-ACACTCCAGCTGGGCTGGAGATATGGAAGAG-3′
	Reverse	5′-CTCAACTGGTGTCGTGGAGTCGGCAATTCAGTTGAGACACAGCT-3′
miR-671-5p	Forward	5′-ACACTCCAGCTGGGAGGAAGCCCTGGAGGGG-3′
	Reverse	5′-CTCAACTGGTGTCGTGGAGTCGGCAATTCAGTTGAGCTCCAGCC-3′
GAPDH	Forward	5′-GCACCGTCAAGGCTGAGAAC-3′
	Reverse	5′-GGATCTCGCTCCTGGAAGATG-3′
U6	Forward	5′-CTCGCTTCGGCAGCACA-3′
	Reverse	5′-AACGCTTCACGAATTTGCGT-3′
AFP	Forward	5′-CTTTGGGCTGCTCGCTATGA-3′
	Reverse	5′-GCATGTTGATTTAACAAGCTGCT-3′
miR-1270 mimics	Sense	5′-CUGGAGAUAUGGAAGAGCUGUGU-3′
	Antisense	5′-ACAGCUCUUCCAUAUCUCCAGUU-3′
miR-1270 inhibitor	Sense	5′-ACACAGCUCUUCCAUAUCUCCAG-3′

### RNase R digestion

5 μg total RNA was incubated with 3 U/μg RNase R (Epicentre Biotechnologies, Shanghai, China) for 15 min at 37°C. The resulting RNAs were subsequently purified using an RNeasy MinElute cleaning Kit (Qiagen, Shanghai, China). After treatment with RNase R, circRNA Cdr1as and GAPDH mRNA expression were detected by qRT-PCR.

### Sanger sequence

Sanger sequencing (authorized to Realgene, Nanjing, China) was performed with the insertion of T vector in the amplified product. Full length was determined, and the back-splice joint of circRNA Cdr1as was verified using the constructed primers (Invitrogen, Shanghai, China).

### CCK8 assay

The proliferation of cells was tested by CCK8 kit (Beyotime, Nantong, China). Approximately transfected 1×10^3^ cells in 100ml were incubated in triplicate in 96-well plates. At 0, 24, 48, 72 and 96h, the CCK-8 reagent (10ml) was added to each well and incubated at 37°C for 2h. Absorbance at 450 nm was recorded by the TECAN infinite M200 Multimode microplate reader (Tecan, Mechelen, Belgium).

### EdU assay

The cell proliferation was tested by EdU assay using Cell-Light EdU DNA Cell Proliferation Kit (RiboBio, Guangzhou, China). After incubation at 37°C and 5% CO_2_ for 48h, transfected cells were added with 50mM EdU and incubated for another 2h. Cells were then fixed with 4% paraformaldehyde and stained with Apollo Dye Solution for proliferating cells. Nucleic acids in all cells were stained with DAPI. The cell proliferation rate was calculated using ImageJ software (version 1.8.0; National Institutes of Health, Sacaton, AZ, USA). Images were taken using a fluorescence microscope.

### Transwell migration assay

100 μL of serum-free suspension was applied in the upper chamber, and 600 μL of medium with 10% FBS was supplied in the bottom chamber. After overnight incubation, cells were subjected to crystal violet (Beyotime, Nantong, China) staining. Penetrating cells were captured and calculated with 5 randomly selected fields per well in triplicate (magnification 200×).

### Dual-luciferase reporter gene assay

Wild-type plasmids circRNA Cdr1as-WT and AFP-WT, and mutant-type plasmids circRNA Cdr1as-MUT and AFP-MUT were inserted into the pGL3 promoter vector (GenePharma, Shanghai, China). HCC cells seeded into 24-well plate were co-transfected with 50 nM miRNAs mimics/negative control and 80 ng plasmid with 5 ng pRL-SV40 using Lipofectamine 3000. Luciferase activity was recorded using the dual-luciferase reporter assay kit (Promega, Madison, WI, USA).

### Chromatin fractionation

We used the PARIS Kit (Life Technologies, USA) to extract cytoplasmic and nuclear RNA, and were further subjected to qRT-RCR, with GAPDH and U6 as the internal references, respectively.

### RIP assay

HCC cells were firstly lysed in RIP lysis buffer. Anti-AGO2 (ab32381, Abcam, Cambridge, MA, USA) or anti-IgG (Millipore, Billerica, MA, USA) conjugated with RNA magnetic beads were subjected to qRT-PCR to determine levels of circRNA Cdr1as and miRNA-1270.

### Exosome extraction

Culture medium was centrifuged at 3000 g for 15 min to remove cells and cellular debris. Exosomes were extracted using the Exoquick exosome precipitation solution (System Biosciences, CA, USA).

### Transmission electron microscopy (TEM)

The extracted exosomes were suspended in 100 μL of PBS and fixed in 5% glutaraldehyde at 4 °C until TEM analysis. A drop of exosome sample on a carbon-coated copper grid was incubated with 2% phosphotungstic acid solution (pH 7.0) for 30 s, and finally observed under a transmission electron microscope (Tecnai G2 Spirit Bio TWIN, FEI, USA).

### Exosome labeling

Exosomes extracted from 1.5 × 10^6^ HCC cells were suspended in 100 μL of PBS, and cultured with 1 ml of mixed PKH67 (Sigma, in Diluent C) for 4 min. 2 ml of 0.5% bovine serum albumin (BSA) was added to terminate exosome labeling, and the extracted exosomes were suspended in 9.6 ml of basal medium. Subsequently, 250 μL of exosome suspension was added to the sub-confluent layer of 293T cells. After 3 h of incubation, cells were washed and fixed at room temperature. To stain the nuclei, 4′,6-diamidino-2-phenylindole (DAPI, Sigma) was added for 10 min, and the stained cells were observed with a fluorescence microscope (Zeiss, LSM700B, Germany).

### Western blot

Protein samples extracted from cells using the Radio Immunoprecipitation Assay (RIPA Beyotime, Nantong, China) buffer were electrophoresed on 10% SDS-PAGE (sodium dodecyl sulphate-polyacrylamide gel electrophoresis), and transferred to PVDF membranes (Millipore, Billerica, MA, USA). After blocking in 5% skim milk for 2 h, membranes were incubated with anti-GAPDH (Beyotime, Nantong, China), anti-TSG101 (ab125011, Abcam), anti-CD63 (ab134045, Abcam) and anti-AFP (ab3980, Abcam) at 4^o^C overnight. 24 h later, membranes were incubated with secondary anti-body (Beyotime, Nantong, China) for 1 h. Band visualization was conducted using the enhanced chemiluminescence reagent kit (Millipore, Billerica, MA, USA).

### HCC samples

HCC tissues and the matched para-carcinoma tissues (n=42) were surgically resected from HCC patients from The Affiliated Huaian No.1 People’s Hospital of Nanjing Medical University or Nantong University Affiliated Hospital and were confirmed by pathological examination. Patients signed written informed consents. This research was approved by the Ethics Committees of both Nantong University Affiliated Hospital and The Affiliated Huaian No.1 People’s Hospital of Nanjing Medical University.

### Fluorescent *in situ* hybridization

FISH was performed as previously described [60]. It is a powerful technique that uses non-toxic fluorescent DNA probes to target any given sequence within a nucleus, resulting in colored signals that are detected with a fluorescence microscope and was performed at Biosense Co. Ltd (Guangzhou, China). Briefly, paraffin-embedded tissues from HCC patients and tumor-bearing nude mice were retrospectively collected. circRNA Cdr1as-positive expression was examined using a FAM-labeled oligonucleotide probe indirectly labeled with FAM-antibody-conjugated quantum dots and miR-1270 was detected using a CY5-labeled oligonucleotide probe.

### Tumourigenicity assay

Animal procedures conformed to the guidelines of the responsible governmental animal ethics committee. 5-week-old nude mice (Shanghai Institute for Biological Sciences) in specific pathogen free level were housed in laminar airflow cabinets. 1 × 10^6^ SMMC-7721 cells stably transfected with circRNA Cdr1as overexpression vector or NC vector suspended in 100 μL Hank’s balanced salt solution were subcutaneously implanted in to mouse back. Tumors were harvested at 4 weeks.

### Lung metastasis assay

Briefly, 1×10^6^ SMMC-7721 cells in 30 μL of 30% Matrigel were injected intravenously through the tail vein of nude mice. After 4 weeks, nude mice were sacrificed, and metastatic nodules in each lung were analyzed. All animal experiments were performed using protocols approved by the animal ethics committee, The Affiliated Huaian No.1 People’s Hospital of Nanjing Medical University.

### H&E staining

Tissues were fixed in 10% formalin, processed and paraffin embedded. Sections in 10 μm thickness were dyed with haematoxylin and eosin for morphological observation.

### Immunohistochemical staining

Paraffin-embedded tissues were incubated with AFP (1:100, Abcam, Cambridge, UK), Ki67 (1:200, Abcam, Cambridge, UK) and PCNA (1:200, Abcam, Cambridge, UK). Slides were dried, dewaxed and rehydrated. DAB (Invitrogen, Carlsbad, CA, USA) substrate chromogen solution was applied, followed by counterstaining with haematoxylin.

### Statistical analysis

SPSS 22.0 software (SPSS Inc., Chicago, IL, USA) was used for data analysis. Data were presented as mean ±SEM. The characteristic differences between Low expression group patients and High expression group patients were assessed by two-side χ^2^ or Fisher's exact test. Two-tailed Student’s *t*-test or Mann-Whitney U-test were performed for assessing the significance of between-group differences. One-way ANOVA followed by Dunnett's multiple comparison test were performed for multigroup comparisons. The Pearson correlation analysis was performed to assess correlations between circRNA Cdr1as, miR-1270 and AFP. *P* < 0.05 was considered as significant.

## Supplementary Material

Supplementary Figures

Supplementary Tables
