# Supplementary material for: CircRNA Cdr1as functions as a competitive endogenous RNA to promote hepatocellular carcinoma progression
Source: Aging (Albany NY). 2019 Oct 1;11(19):8182–203. doi: 10.18632/aging.102312 (PMC6814590; doi:10.18632/aging.102312)
Supplement: Supplementary Figures [file aging-11-102312-s002.pdf]

SUPPLEMENTARY FIGURES

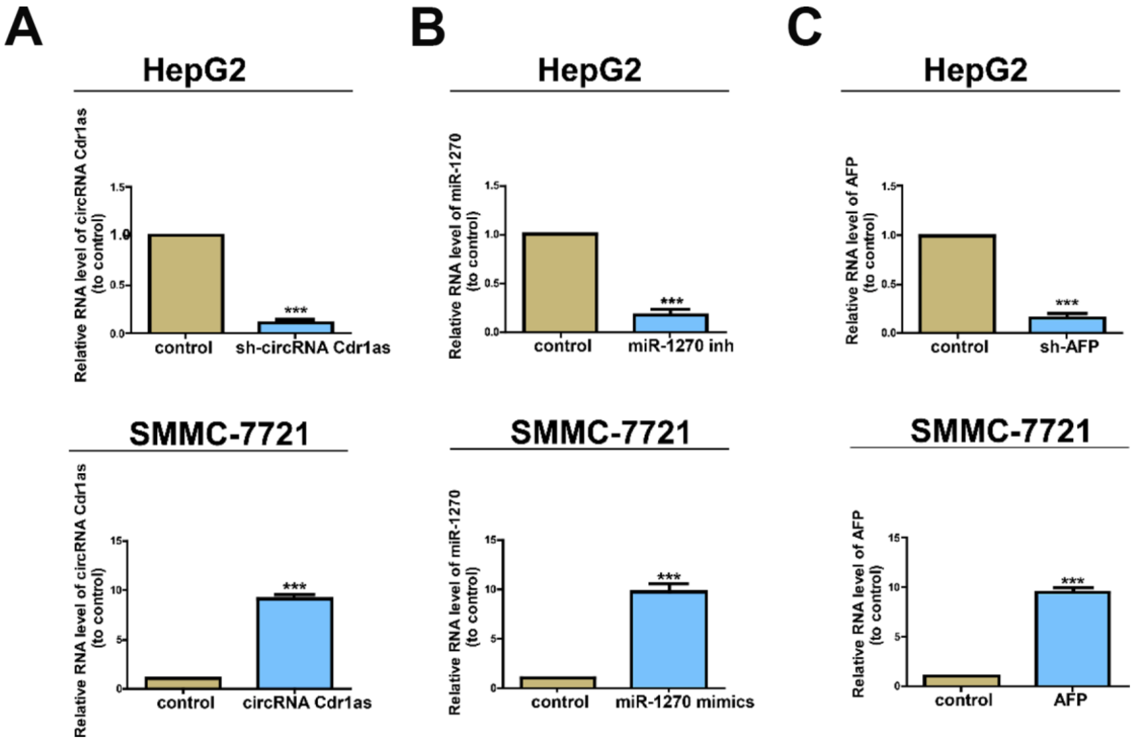

Supplementary Figure 1. (**A**) After transfection, the relative levels of circRNA Cdr1as were detected by qRT-PCR. (**B**) After transfection, the relative levels of miR-1270 were detected by qRT-PCR. (**C**) After transfection, the relative levels of AFP were detected by qRT-PCR. Results are presented as mean ± SD. **P*<0.05, ***P*<0.01, ****P*<0.001. All of the experiments were performed in triplicate.

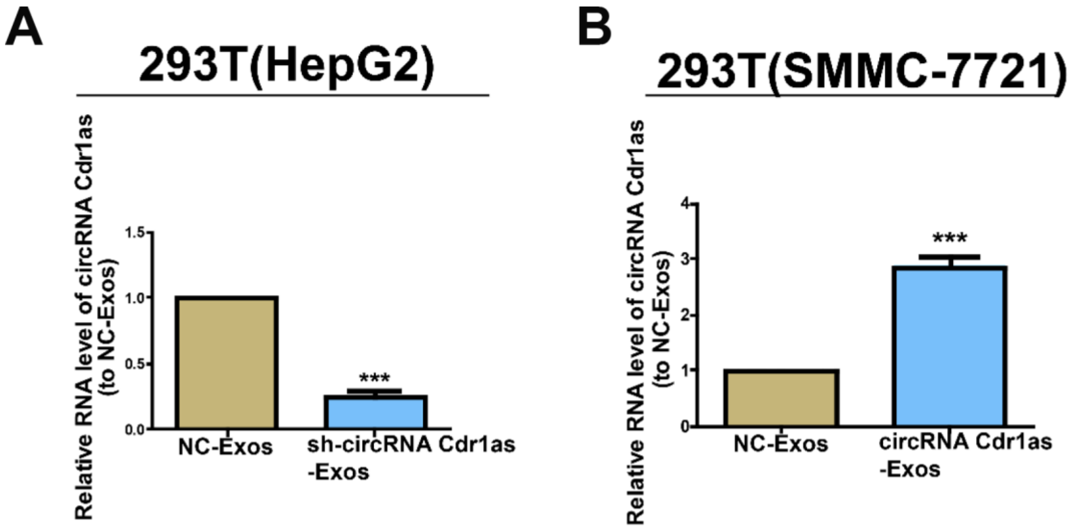

Supplementary Figure 2. (**A**) The levels of circRNA Cdr1as in HepG2, SMMC-7721 and 293T cells. (**B**) The levels of exosomal circRNA Cdr1as in HepG2, SMMC-7721 and 293T cells. Results are presented as mean ± SD. **P*<0.05, ***P*<0.01, ****P*<0.001. All of the experiments were performed in triplicate.

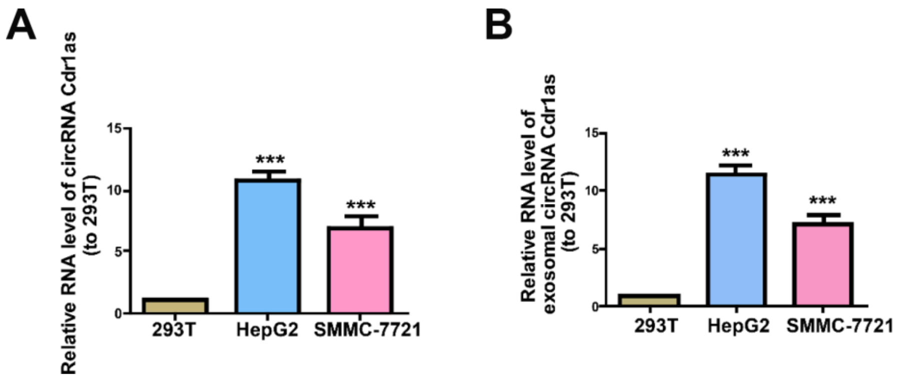

Supplementary Figure 3. (**A**) Comparing with control group, circRNA Cdr1as level in 293T cells was reduced by exposure of sh-circRNA Cdr1as-Exos. (**B**) Comparing with control group, circRNA Cdr1as level in 293T cells exposed to circRNA Cdr1as-Exos was elevated. Results are presented as mean ± SD. **P*<0.05, ***P*<0.01, ****P*<0.001. All of the experiments were performed in triplicate.
