# Supplementary material for: CircRNA Cdr1as functions as a competitive endogenous RNA to promote hepatocellular carcinoma progression
Source: Aging (Albany NY). 2019 Oct 1;11(19):8182–203. doi: 10.18632/aging.102312 (PMC6814590; doi:10.18632/aging.102312)
Supplement: Supplementary Tables [file aging-11-102312-s001.pdf]

SUPPLEMENTARY TABLES

Supplementary Table 1. Relationship between circRNA Cdr1as expression and clinicopathological characteristics of hepatocellular carcinoma patients.

Clinicopathological characteristics	n	High expression	Low expression	P value
**Total**	42	21	21	
**Gender**				1.000
Male	30	15	15	
Female	12	6	6	
**Age (years)**				0.758
≤54	20	11	9	
>54	22	10	12	
**Grade of differentiation**				0.589
Low	17	9	8	
Middle	15	6	9	
High	10	6	4	
**Tumor diameter (cm)**				**0.012***
≤5	19	5	14	
>5	23	16	7	
**Liver function (Child-Pugh stage)**				1.000
A	35	17	18	
B or C	7	4	3	
**Hepatocirrhosis**				0.719
Absent	10	6	4	
Present	32	15	17	
**HBV infection**				0.520
Absent	15	9	6	
Present	27	12	15	
**AFP (ng/ml)**				**0.005***
≤20	20	5	15	
>20	22	16	6	
**Tumor satellite**				**0.033***
Absent	31	12	19	
Present	11	9	2	

Two-sided χ^2 or Fisher's exact test for all variables between Low expression group and High expression group, *P<0.

Supplementary Table 2. The potential miRNAs binding to circRNA Cdr1as through bioinformatics prediction.

RegRNA	Circular RNA Interactome	Intersection
hsa-miR-7-5p	hsa-miR-1180	**hsa-miR-671-5p**
**hsa-miR-671-5p**	hsa-miR-1231	**hsa-miR-1270**
**hsa-miR-1270**	hsa-miR-1243	
hsa-miR-3156-5p	hsa-miR-1246	
	hsa-miR-1277	
	hsa-miR-1287	
	hsa-miR-1290	
	hsa-miR-1299	
	hsa-miR-1304	
	hsa-miR-203	
	hsa-miR-21	
	hsa-miR-590-5p	
	hsa-miR-432	
	hsa-miR-490-5p	
	hsa-miR-516b	
	hsa-miR-576-3p	
	hsa-miR-619	
	hsa-miR-1270	
	hsa-miR-620	
	hsa-miR-647	
	hsa-miR-671-5p	
	hsa-miR-7	
	hsa-miR-873	
	hsa-miR-890	
	hsa-miR-944	
